# Improved Handwritten Digit Recognition Using Convolutional Neural Networks (CNN)

**DOI:** 10.3390/s20123344

**Published:** 2020-06-12

**Authors:** Savita Ahlawat, Amit Choudhary, Anand Nayyar, Saurabh Singh, Byungun Yoon

**Affiliations:** 1 Department of Computer Science and Engineering, Maharaja Surajmal Institute of Technology, New Delhi 110058, India; savita.ahlawat@gmail.com; 2 Department of Computer Science, Maharaja Surajmal Institute, New Delhi 110058, India; amit.choudhary69@gmail.com; 3 Graduate School, Duy Tan University, Da Nang 550000, Vietnam; anandnayyar@duytan.edu.vn; 4 Department of Industrial & Systems Engineering, Dongguk University, Seoul 04620, Korea; saurabh89@dongguk.edu

**Keywords:** convolutional neural networks, handwritten digit recognition, pre-processing, OCR

## Abstract

Traditional systems of handwriting recognition have relied on handcrafted features and a large amount of prior knowledge. Training an Optical character recognition (OCR) system based on these prerequisites is a challenging task. Research in the handwriting recognition field is focused around deep learning techniques and has achieved breakthrough performance in the last few years. Still, the rapid growth in the amount of handwritten data and the availability of massive processing power demands improvement in recognition accuracy and deserves further investigation. Convolutional neural networks (CNNs) are very effective in perceiving the structure of handwritten characters/words in ways that help in automatic extraction of distinct features and make CNN the most suitable approach for solving handwriting recognition problems. Our aim in the proposed work is to explore the various design options like number of layers, stride size, receptive field, kernel size, padding and dilution for CNN-based handwritten digit recognition. In addition, we aim to evaluate various SGD optimization algorithms in improving the performance of handwritten digit recognition. A network’s recognition accuracy increases by incorporating ensemble architecture. Here, our objective is to achieve comparable accuracy by using a pure CNN architecture without ensemble architecture, as ensemble architectures introduce increased computational cost and high testing complexity. Thus, a CNN architecture is proposed in order to achieve accuracy even better than that of ensemble architectures, along with reduced operational complexity and cost. Moreover, we also present an appropriate combination of learning parameters in designing a CNN that leads us to reach a new absolute record in classifying MNIST handwritten digits. We carried out extensive experiments and achieved a recognition accuracy of 99.87% for a MNIST dataset.

## 1. Introduction

In the current age of digitization, handwriting recognition plays an important role in information processing. A lot of information is available on paper, and processing of digital files is cheaper than processing traditional paper files. The aim of a handwriting recognition system is to convert handwritten characters into machine readable formats. The main applications are vehicle license-plate recognition, postal letter-sorting services, Cheque truncation system (CTS) scanning and historical document preservation in archaeology departments, old documents automation in libraries and banks, etc. All these areas deal with large databases and hence demand high recognition accuracy, lesser computational complexity and consistent performance of the recognition system. It has been suggested that deep neural architectures are more advantageous than shallow neural architectures [1,2,3,4,5,6]. The key differences are described in Table 1. The deep learning field is ever evolving, and some of its variants are autoencoders, CNNs, recurrent neural networks (RNNs), recursive neural networks, deep belief networks and deep Boltzmann machines. Here, we introduce a convolutional neural network, which is a specific type of deep neural network having wide applications in image classification, object recognition, recommendation systems, signal processing, natural language processing, computer vision, and face recognition. The ability to automatically detect the important features of an object (here an object can be an image, a handwritten character, a face, etc.) without any human supervision or intervention makes them (CNNs) more efficient than their predecessors (Multi layer perceptron (MLP), etc.). The high capability of hierarchical feature learning results in a highly efficient CNN.

A convolutional neural network (CNN) is basically a variation of a multi-layer perceptron (MLP) network and was used for the first time in 1980 [7]. The computing in CNN is inspired by the human brain. Humans perceive or identify objects visually. We (humans) train our children to recognize objects by showing him/her hundreds of pictures of that object. This helps a child identify or make a prediction about objects he/she has never seen before. A CNN works in the same fashion and is popular for analyzing visual imagery. Some of the well-known CNN architectures are GoogLeNet (22 layers), AlexNet (8 layers), VGG (16–19 Ali), and ResNet (152 layers). A CNN integrates the feature extraction and classification steps and requires minimal pre-processing and feature extraction efforts. A CNN can extract affluent and interrelated features automatically from images. Moreover, a CNN can provide considerable recognition accuracy even if there is only a little training data available.

Design particulars and previous knowledge of features are no longer required to be collected. Exploitation of topological information available in the input is the key benefit of using a CNN model towards delivering excellent recognition results. The recognition results of a CNN model are also independent of the rotation and translation of input images. Contrary to this, thorough topological knowledge of inputs is not exploited in MLP models. Furthermore, for a complex problem, MLP is not found to be appropriate, and they do not scale well for higher resolution images because of the full interconnection between nodes, also called the famous phenomenon of the “curse of dimensionality”.

In the past few years, the CNN model has been extensively employed for handwritten digit recognition from the MNIST benchmark database. Some researchers have reported accuracy as good as 98% or 99% for handwritten digit recognition [8]. An ensemble model has been designed using a combination of multiple CNN models. The recognition experiment was carried out for MNIST digits, and an accuracy of 99.73% was reported [9]. Later, this “7-net committee” was extended to the “35-net committee” experiment, and the improved recognition accuracy was reported as 99.77% for the same MNIST dataset [10]. An extraordinary recognition accuracy of 99.81% was reported by Niu and Suen by integrating the SVM (support vector machine) capability of minimizing the structural risk and the capability of a CNN model for extracting the deep features for the MNIST digit recognition experiment [11]. The bend directional feature maps were investigated using CNN for in-air handwritten Chinese character recognition [12]. Recently, the work of Alvear-Sandoval et al. achieved a 0.19% error rate for MNIST by building diverse ensembles of deep neural networks (DNN) [13]. However, on careful investigation, it has been observed that the high recognition accuracy of MNIST dataset images is achieved through ensemble methods only. Ensemble methods help in improving the classification accuracy but at the cost of high testing complexity and increased computational cost for real-world application [14].

The purpose of the proposed work is to achieve comparable accuracy using a pure CNN architecture through extensive investigation of the learning parameters in CNN architecture for MNIST digit recognition. Another purpose is to investigate the role of various hyper-parameters and to perform fine-tuning of hyper-parameters which are essential in improving the performance of CNN architecture.

Therefore, the major contribution of this work is in two respects. First, a comprehensive evaluation of various parameters, such as numbers of layers, stride size, kernel size, padding and dilution, of CNN architecture in handwritten digit recognition is done to improve the performance. Second, optimization of the learning parameters achieved excellent recognition performance on the MNIST dataset. The MNIST database has been used in this work because of the availability of its published results with different classifiers. The database is also popular and mostly used as a benchmark database in comparative studies of various handwritten digit recognition experiments for various regional and international languages.

The novelty of the proposed work lies in the thorough investigation of all the parameters of CNN architecture to deliver the best recognition accuracy among peer researchers for MNIST digit recognition. The recognition accuracy delivered in this work employing a fine-tuned pure CNN model is superior to the recognition accuracies reported by peer researchers using an ensemble architecture. The use of ensemble architecture by peer researchers involves increased computational cost and high testing complexity. Hence, the proposed pure CNN model outperforms the ensemble architecture offered by peer researchers both in terms of recognition accuracy as well as computational complexity.

The rest of the paper is organized as follows: Section 2 describes the related work in the field of handwriting recognition; Section 3 and Section 4 describe CNN architecture and the experimental setup, respectively; Section 5 discusses the findings and presents a comparative analysis; and Section 6 presents the conclusion and suggestions for future directions.

## 2. Related Work

Handwriting recognition has already achieved impressive results using shallow networks [15,16,17,18,19,20,21,22,23,24]. Many papers have been published with research detailing new techniques for the classification of handwritten numerals, characters and words. The deep belief networks (DBN) with three layers along with a greedy algorithm were investigated for the MNIST dataset and reported an accuracy of 98.75% [25]. Pham et al. applied a regularization method of dropout to improve the performance of recurrent neural networks (RNNs) in recognizing unconstrained handwriting [26]. The author reported improvement in RNN performance with significant reduction in the character error rate (CER) and word error rate (WER).

The convolutional neural network brings a revolution in the handwriting recognition field and delivered the state-of-the-art performance in this domain [27,28,29,30,31,32]. In 2003, Simard et al. introduced a general convolutional neural network architecture for visual document analysis and weeded out the complex method of neural network training [33]. Wang et al. proposed a novel approach for end-to-end text recognition using multi-layer CNNs and achieved excellent performance on benchmark databases, namely, ICDAR 2003 and Street View Text [34]. Recently, Shi et al. integrated the advantages of both the deep CNN (DCNN) and recurrent neural network (RNN) and named it conventional recurrent neural network (CRNN). They applied CRNN for scene text recognition and found it to be superior to traditional methods of recognition [35]. Badrinarayanan et al. proposed a deep convolution network architecture for semantic segmentation. The segmentation architecture is known as SegNet and consists of an encoder network, a decoder network and a pixel-wise classification layer. The proposed method used max-pooling indices of a feature map while decoding and observed good performance. The method is also analyzed and compared with existing techniques for road scene and indoor understanding [36,37,38]. CNN has shown remarkable abilities in offline handwritten character recognition of Arabic language [39]; handwritten Tamil character recognition [40]; Telugu character recognition [41], handwritten Urdu text recognition [42,43], handwritten character recognition in Indic scripts [44] and Chinese handwritten text recognition [45,46,47].

Recently, Gupta et al. in [48] proposed a novel multi-objective optimization framework for identifying the most informative local regions from a character image. The work was also evaluated on isolated handwritten English numerals, namely, MNIST images, along with three other popular Indic scripts, namely, handwritten Bangala numerals and handwritten Devanagari characters. The authors used features extracted from a convolutional neural network in their model and achieved 95.96% recognition accuracy. The work of Nguyen et al. in [49] used a multi-scale CNN for extracting spatial classification features for handwritten mathematical expression (HME). The local features and spatial information of HME images were used for clustering HME images. The work observed high performance for the CROHME dataset. They (authors) also concluded that classification can be improved by training the CNN with a combination of global max pooling and global attentive pooling. Ziran et al. [50] developed a faster R-CNN-based framework for text/word location and recognition in historical books. The authors evaluated these deep learning methods on Gutenberg’s Bible pages. The handwritten character recognition problem is intelligently addressed in the work of Ptucha et al. [51] by the introduction of an intelligent character recognition (ICR) system using a conventional neural network. The work was evaluated on French-based RIMES lexicon datasets and English-based IAM datasets, showing substantial improvement.

The performance of CNNs depends mainly on the choice of hyper-parameters [52], which are usually decided on a trial-and-error basis. Some of the hyper-parameters are, namely, activation function, number of epochs, kernel size, learning rate, hidden units, hidden layers, etc. These parameters are very important as they control the way an algorithm learns from data [53]. Hyper-parameters differ from model parameters and must be decided before the training begins.

ResNet-52 [54], GoogleNet [55], VGG-16 [56] and AlexNet [57] are some popular CNN models that have a total of 150, 78, 57 and 27 hyper-parameters, respectively. A bad choice for hyper-parameters can incur a high computation cost and lead to poor CNN performance. The researcher’s expertise plays an important role in deciding on the configuration of hyper-parameters and requires an intelligent strategic plan. This creates several questions about CNN design for handwriting recognition tasks. How is CNN better in extracting distinct features from handwritten characters? What effect do different hyper-parameters have on CNN performance? What is the role of design parameters in improving CNN performance? In order to guide future research in the handwriting recognition field, it is important to address these questions.

## 3. Convolutional Neural Network Architecture

**Figure 1:**
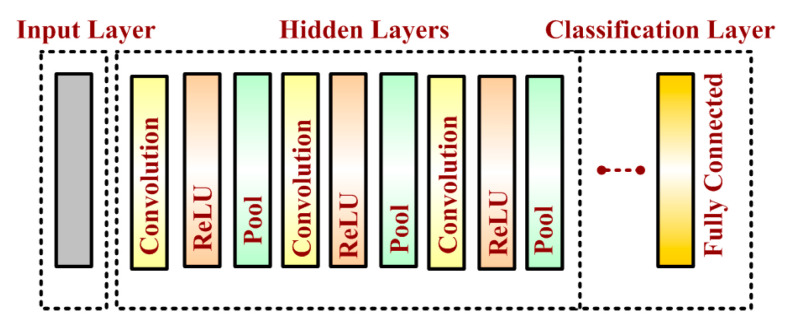
Typical convolutional neural network architecture.

A basic convolutional neural network comprises three components, namely, the convolutional layer, the pooling layer and the output layer. The pooling layer is optional sometimes. The typical convolutional neural network architecture with three convolutional layers is well adapted for the classification of handwritten images as shown in Figure 1. It consists of the input layer, multiple hidden layers (repetitions of convolutional, normalization, pooling) and a fully connected and an output layer. Neurons in one layer connect with some of the neurons present in the next layer, making the scaling easier for the higher resolution images. The operation of pooling or sub-sampling can be used to reduce the dimensions of the input. In a CNN model, the input image is considered as a collection of small sub-regions called the “receptive fields”. A mathematical operation of the convolution is applied on the input layer, which emulates the response to the next layer. The response is basically a visual stimulus. The detailed description is as follows:

### 3.1. Input Layer

The input data is loaded and stored in the input layer. This layer describes the height, width and number of channels (RGB information) of the input image.

### 3.2. Hidden Layer

The hidden layers are the backbone of CNN architecture. They perform a feature extraction process where a series of convolution, pooling and activation functions are used. The distinguishable features of handwritten digits are detected at this stage.

### 3.3. Convolutional Layer

The convolutional layer is the first layer placed above the input image. It is used for extracting the features of an image. The *n* × *n* input neurons of the input layer are convoluted with an *m* × *m* filter and in return deliver (*n* − *m* + 1) × (*n* − *m* + 1) as output. It introduces non-linearity through a neural activation function. The main contributors of the convolutional layer are receptive field, stride, dilation and padding, as described in the following paragraph.

**Figure 2:**
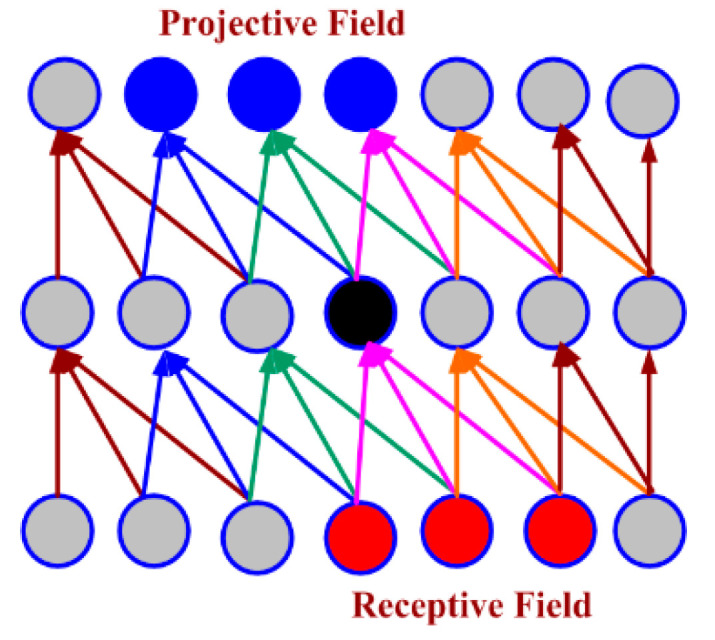
Receptive field and projective field.

**Figure 3:**
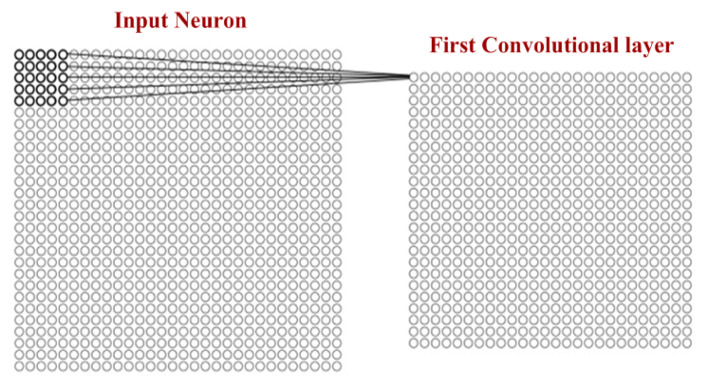
Visualization of filter of size 5 × 5 with activation map. (Input neuron 28 × 28 and convolutional layer 24 × 24).

**Figure 4:**
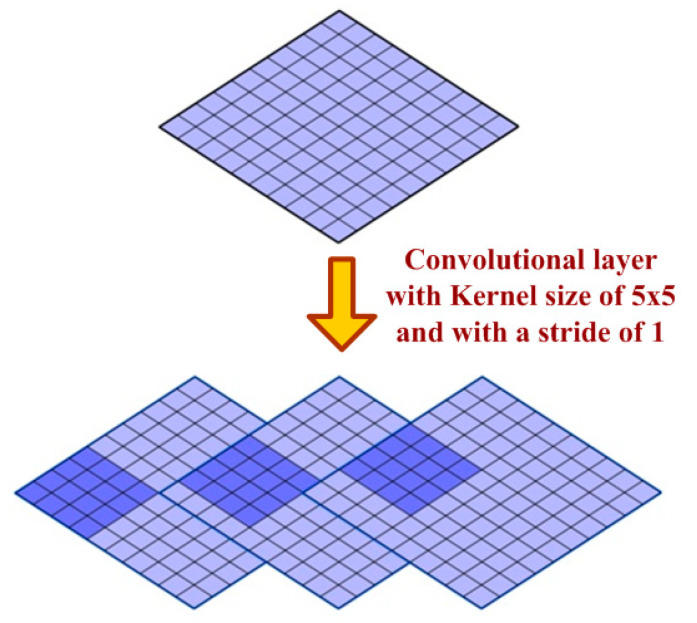
Representation of kernel and stride in a convolutional layer.

CNN computation is inspired by the visual cortex in animals [58]. The visual cortex is a part of the brain that processes the information forwarded from the retina. It processes visual information and is subtle to small sub-regions of the input. Similarly, a receptive field is calculated in a CNN, which is a small region of an input image that can affect a specific region of the network. It is also one of the important design parameters of the CNN architecture and helps in setting other CNN parameters [59]. It has the same size as the kernel and works in a similar fashion as the foveal vision of the human eye works for producing sharp central vision. The receptive field is influenced by striding, pooling, kernel size and depth of the CNN [60]. Receptive field (r), effective receptive field (ERF) and projective field (PF) are terminology used in calculating effective sub-regions in a network. The area of the original image influencing the activation of a neuron is described using the ERF, whereas the PF is a count of neurons to which neurons project their outputs, as described in Figure 2. The visualization of the 5 × 5-size filter and its activation map are described in Figure 3. Stride is another parameter used in CNN architecture. It is defined as the step size by which the filter moves every time. A stride value of 1 indicates the filter sliding movement pixel by pixel. A larger stride size shows less overlapping between the cells. The working of kernel and stride in the convolution layer is presented in Figure 4.

The concept of padding is introduced in CNN architecture to get more accuracy. Padding is introduced to control the shrinking of the output of the convolutional layer.

The output from the convolutional layer is a feature map, which is smaller than the input image. The output feature map contains more information on middle pixels and hence loses lots of information present on corners. The rows and the columns of zeros are added to the border of an image to prevent shrinking of the feature map.

Equations (1) and (2) describe the relationship between the size of the feature map, the kernel size and stride while calculating the size of the output feature map.

(1) $$W_{nx}=W_{n-1x}-F_{nx}S_{nx}+1$$

(2) $$W_{ny}=W_{n-1y}-F_{ny}S_{ny}+1$$

where ($W_{nx},W_{ny})$ represent the size of the output feature map, ($S_{nx},S_{ny})$ is stride size, and ($F_{x},F_{y})$ is kernel size. Here ‘*n*’ is used to describe the index of layers.

**Figure 5:**
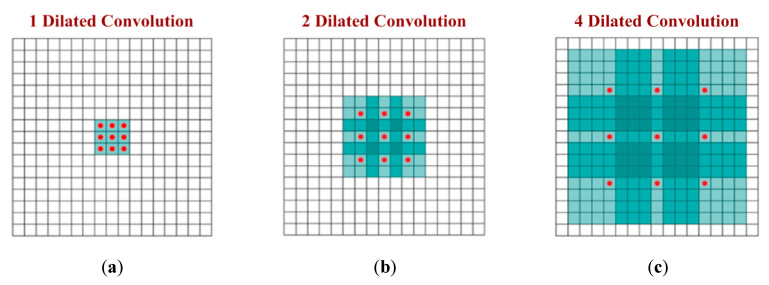
Dilated convolution: (**a**) receptive field of 3 × 3 using 1-dilated convolution; (**b**) receptive field of 7 × 7 using 2-dilated convolution; (**c**) receptive field of 15 × 15 using 4-dilated convolution.

The dilation is another important parameter of CNN architecture that has a direct influence on the receptive field. The dilation can increase the field-of-view (FOV) of a CNN without modifying the feature map [61]. Figure 5 clearly shows that dilation values can exponentially raise the receptive field of a CNN. Too large a dilation can increase the number of computations and hence can slow down the system by increasing the processing time. Therefore, it must be chosen wisely. The relationship between dilation, weight and input is shown in Equations (3) and (4) below.

(3) 0−dialation=w[0]∗x[0]+w[1]∗x[1]+w[2]∗x[2];

(4) 1−dialation=w[0]∗x[0]+w[1]∗x[2]+w[2]∗x[4];

### 3.4. Pooling Layer

**Figure 6:**
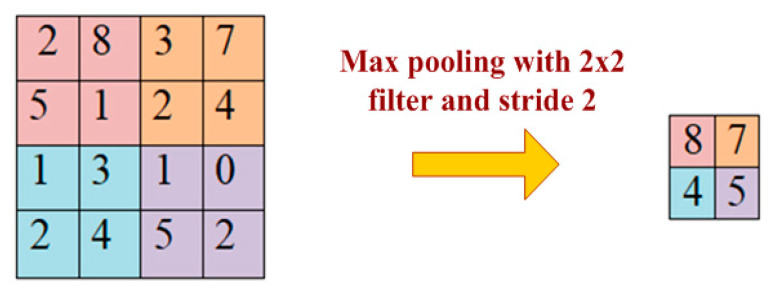
Max pooling with filter 2 × 2 and stride size.

A pooling layer is added between two convolutional layers to reduce the input dimensionality and hence to reduce the computational complexity. Pooling allows the selected values to be passed to the next layer while leaving the unnecessary values behind. The pooling layer also helps in feature selection and in controlling overfitting. The pooling operation is done independently. It works by extracting only one output value from the tiled non-overlapping sub-regions of the input images. The common types of pooling operations are max-pooling and avg-pooling (where max and avg represent maxima and average, respectively). The max-pooling operation is generally favorable in modern applications, because it takes the maximum values from each sub-region, keeping maximum information. This leads to faster convergence and better generalization [62]. The max-pooling operation for converting a 4 × 4 convolved output into a 2 × 2 output with stride size 2 is described in Figure 6. The maximum number is taken from each convolved output (of size 2 × 2) resulting in reducing the overall size to 2 × 2.

### 3.5. Activation Layer

Just like regular neural network architecture, CNN architecture also contains the activation function to introduce the non-linearity in the system. The sigmoid function, rectified linear unit (ReLu) and Softmax are some famous choices among various activation functions exploited extensively in deep learning models. It has been observed that the sigmoid activation function might weaken the CNN model because of the loss of information present in the input data. The activation function used in the present work is the non-linear rectified linear unit (ReLu) function, which has output 0 for input less than 0 and raw output otherwise. Some advantages of the ReLu activation function are its similarity with the human nerve system, simplicity in use and ability to perform faster training for larger networks.

### 3.6. Classification Layer

The classification layer is the last layer in CNN architecture. It is a fully connected feed forward network, mainly adopted as a classifier. The neurons in the fully connected layers are connected to all the neurons of the previous layer. This layer calculates predicted classes by identifying the input image, which is done by combining all the features learned by previous layers. The number of output classes depends on the number of classes present in the target dataset. In the present work, the classification layer uses the ‘softmax’ activation function for classifying the generated features of the input image received from the previous layer into various classes based on the training data.

### 3.7. Gradient Descent Optimization Algorithm

Optimization algorithms are used to optimize neural networks and to generate better performance and faster results. The algorithm helps in minimizing or maximizing a cost function by updating the weight/bias values, which are known as learning parameters of a network, and the algorithm updating these values is termed as the adaptive learning algorithm. These learning parameters directly influence the learning process of a network and have an important role in producing an efficient network model. The aim of all the optimization algorithms is to find the optimum values of these learning parameters. The gradient descent algorithm is one such optimization algorithm. Recent classification experiments based on deep learning reported excellent performance with the progress in learning parameter identification [63,64,65,66,67,68,69].

Gradient descent, as the name implies, uses an error gradient to descend along with the error surface. It also allows a minimum of a function to be found when a derivative of it exists and there is only one optimum solution (if we expect local minima). The gradient is the slope of the error surface and gives an indication of how sensitive the error is towards the change in the weights. This sensitivity can be exploited for incremental change in the weights towards the optimum. Gradient descent is classified into three types, namely, batch gradient descent, stochastic gradient descent (SGD) and mini-batch gradient descent.

The SGD algorithm works faster and has been extensively used in deep learning experiments [70,71,72,73,74]. The SGD algorithm avoids redundant computation better than the batch gradient descent algorithm. The algorithm steps for updating the learning parameter for each training data bit are as follows (Algorithm 1):

**Algorithm 1** Learning Parameters Update Mechanism
1: **Input:**
(x(i), y(i)) as training data; η as learning rate; ∇E(θ) is a gradient of loss (error) function E(θ) with respect to the θ parameter; momentum factor (m) 2: **Output:** For each training pair, update the learning parameters using the equation *θ*=*θ*-η.∇E(*θ*;x(i);y(i)) 3: **if** stopping condition is met 4: **return** parameter *θ*.

The SGD algorithm can be further optimized using various optimizers, such as momentum, adaptive moment estimation (Adam), Adagrad and Adadelta. The momentum parameter is selected to speed up SGD optimization. The momentum optimizer can reduce the unnecessary parameter update, which leads to faster convergence of the network. The modified update equations with the momentum factor (m) are given by (5).

(5) $$\begin{array}{c} z\left(t\right)=mz\left(t-1\right)+\eta \nabla E\left(\theta \right)\\ \theta =\theta -z\left(t\right) \end{array}\}$$

Most implementation usually considers m=0.9 for updating the learning parameters.

Adagrad is one of the SGD algorithms widely preferred in optimizing sparse data. The Adagrad optimizer works by considering the past gradients and modifying the learning rate for every parameter at each time step. The update equation for the Adagrad optimizer is given by (6).

(6) $$\theta_{t+1,i}=\theta_{t,i}-\frac{\eta }{\sqrt{G_{t,\;ii}+\varepsilon }}\;.g_{t,i}$$

where g(t,i) is a gradient of the loss function for parameter θ(i) at a time step *t*; ε is a smoothing term taken to avoid the division by 0; and $G_{t,\;ii}$ is a diagonal matrix representing the sum of gradient squares for parameter θ(i) for a time step *t*;

The learning rate in the Adagrad optimizer keeps on decreasing, which causes a slow convergence rate and longer training time. Adadelta is another adaptive learning algorithm and is an extension of the Adagrad optimizer. It can beat the decaying learning rate problem of the previous optimizer, i.e., Adagrad [61]. Adadelta limits the storage of past squared gradients by fixing the storage window size. The moving averages of the square of gradients and square of weights updates are calculated. The learning rate is basically the square root of the ratio between these moving averages. A running average $E{\left[g^{2}\right]}_{t}$ at time step *t* of the past gradient is calculated using (7).

(7) $$E{\left[g^{2}\right]}_{t}=\gamma E{\left[g^{2}\right]}_{t-1}+\left(1-\gamma \right)g_{t}^{2}$$

where I_ is similar to the momentum term.

The parameter update for Adadelta optimization is done using Equations (8) and (9).

(8) $$\Delta \theta_{t}=-\eta .g_{t,i}$$

(9) $$\Delta \theta_{t+1}=\theta_{t}+\Delta \theta_{t}$$

or (10) can be modified as

(10) $$\Delta \theta_{t}=-\frac{\eta }{\sqrt{G_{t}+\varepsilon }}⊙g_{t}$$

Replacing the diagonal matrix with the running average $E{\left[g^{2}\right]}_{t}$,

(11) $$\Delta \theta_{t}=-\frac{\eta }{\sqrt{E{\left[g^{2}\right]}_{t}+\varepsilon }}⊙g_{t}$$

(12) $$\Delta \theta_{t}=-\frac{\eta }{RMS{\left[g\right]}_{t}}⊙g_{t}$$

where $RMS{\left[g\right]}_{t}$ is the parameter update root mean square error and is calculated using (13):

(13) $$RMS{\left[g\right]}_{t}=\sqrt{E{\left[g^{2}\right]}_{t}+\varepsilon }$$

Adam is another famous SGD optimizer having learning weight updating similar to the storing average in Adadelta and decaying average of past squared gradients as present in the momentum optimizer. Adam outperforms other techniques by performing fast convergence with a fast learning speed. Equations (14) and (15) are used to calculate the first-moment value (M(t)) and the variance of gradients (v(t)):

(14) $${\hat{m}}_{t}=\frac{m_{t}}{1-\beta_{1}^{t}}$$

(15) $${\hat{v}}_{t}=\frac{v_{t}}{1-\beta_{2}^{t}}$$

and the parameter update is given in Equation (16):

(16) $$\theta_{t+1}=\theta_{t}-\frac{\eta }{\sqrt{{\hat{v}}_{t}+\varepsilon }}{\hat{m}}_{t}$$

## 4. Experimental Setup

To accomplish the task of handwritten digit recognition, a model of the convolutional neural network is developed and analyzed for suitable different learning parameters to optimize recognition accuracy and processing time. We propose to investigate variants of CNN architecture with three layers (CNN_3L) and variants of CNN architecture with four layers (CNN_4L). A total of six cases (case 1 to case 6) have been considered for CNN with three-layer architecture and five cases (case 1 to case 5) for CNN architecture with four layers. All the cases differ in the number of feature maps, stride sizes, padding, dilation and received receptive fields.

The recognition process of the handwritten digits consists of the following steps:

1. To acquire or collect the MNIST handwritten digit images.
2. To divide the input images into training and test images.
3. To apply the pre-processing technique to both the training dataset and the test dataset.
4. To normalize the data so that it ranges from 0 to 1.
5. To divide the training dataset into batches of a suitable size.
6. To train the CNN model and its variants using the labelled data.
7. To use a trained model for the classification.
8. To analyze the recognition accuracy and processing time for all the variants.

In summary, the present work for handwritten digit recognition investigates the role of training parameters, gradient descent optimizers and CNN architecture.

**Figure 7:**
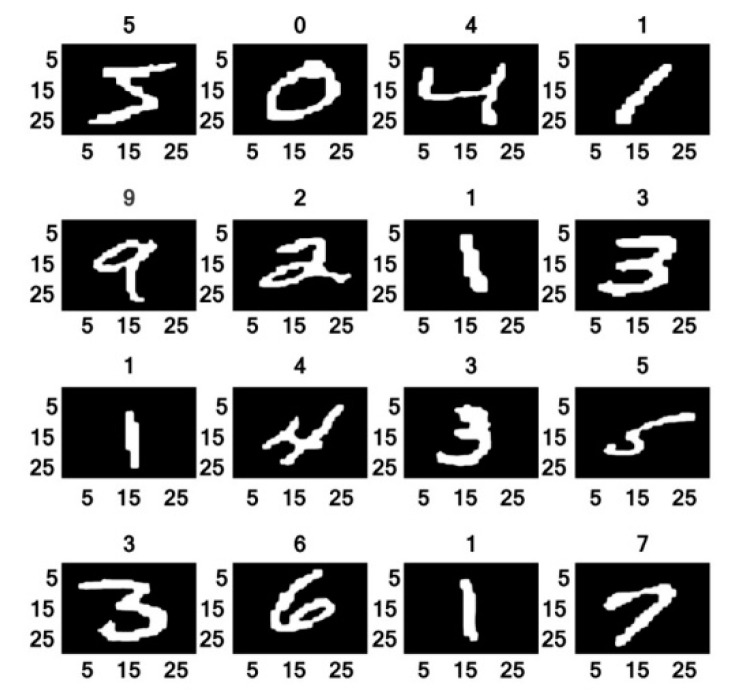
Sample MNIST handwritten digit images.

All the experiments were done in MATLAB 2018b on a personal computer with Windows 10, Intel (R) Core (TM) i7-6500 CPU (2.50 GHz), 16.00 GB memory and NVIDIA 1060 GTX GPU. The MNIST database was involved in the training and testing models. The standard MNIST handwritten digit database has 60,000 and 10,000 normalized digit images in its training and testing datasets, respectively. Some of the sample images from the MNIST database are shown in Figure 7.

Data pre-processing plays an important role in any recognition process. To shape the input images in a form suitable for segmentation, the pre-processing methods, such as scaling, noise reduction, centering, slanting and skew estimation, were used. In general, many algorithms work better after the data has been normalized and whitened. One needs to work with different algorithms in order to find out the exact parameters for data pre-processing. In the present work, the MNIST dataset images were size-normalized into a fixed image of size 28 × 28.

A convolution layer with 28 kernel/filter/patches and kernel sizes of 5 × 5 and 3 × 3 is used here to extract the features. Every patch contains the structural information of an image. For example, a convolution operation is computed by sliding a filter of size 5 × 5 over input image. This layer is responsible for transforming the input data by using a patch/filter of locally connecting neurons from the previous layer.

## 5. Results and Discussion

The experimental results of the MNIST handwritten digit dataset using different parameters of CNN_3L and CNN_4L architectures are recorded and analyzed in Table 2 and Table 3, respectively. An MNIST sample image is represented as a 1-D array of 784 (28 × 28) float values between 0 and 1 (0 stands for black, 1 for white). The receptive field (r) is calculated based on kernel size (k), stride (s), dilation (d), padding (p), input size (i/p) and output size (o/p) of the feature map, and the recognition accuracy and total time elapsed is shown in Table 2 and Table 3, employing CNN architecture with three and four layers respectively. The findings of Table 2 confirm the role of different architecture parameters on the performance of our recognition system. The training parameter used here has a learning rate of 0.01 and maximum epoch counts of 4. The highest recognition accuracy achieved in case 3, with CNN_3L architecture having three layers, is 99.76% for the feature map 12-24-32. In case 5, the CNN_4L architecture with four layers achieved the highest recognition accuracy of 99.76 % for the feature map 12-24-28-32, as shown in Table 3.

**Figure 8:**
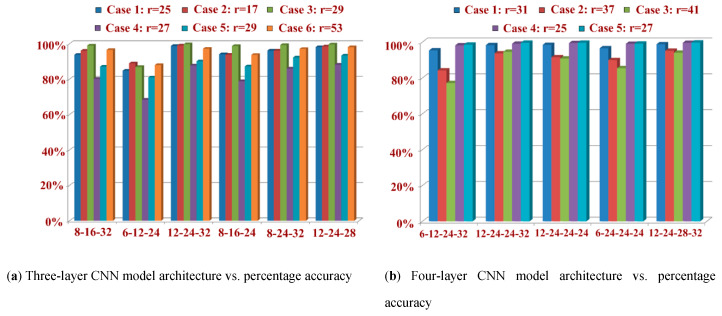
Receptive fields and recognition accuracies for CNN: (**a**) architecture having three layers; (**b**) architecture having four layers.

The values of different architectural parameters have been chosen in such a way as to observe the role of all the parameters. For all convolutional layers, the hyper-parameters include kernel size (1–5), stride (1–3), dilation (1–2) and padding (1–2). The first observation from both of the tables shows the role of the receptive field. The value of the receptive field, when close to the input size, observed good recognition accuracy for all the feature maps (case 3 in Table 2 and case 5 in Table 3). On the other hand, a large gap between receptive field and input size observed poor recognition accuracy (case 3 and case 6 in Table 2 and case 3 in Table 3). The plots of Figure 8a,b clearly describe the relationship between recognition accuracy and receptive field. This highlights that receptive field can easily capture the elementary information like edges and corners from the input images in the lower layers of the CNN, which is passed to the subsequent layers for further processing. From Table 2 and Table 3, it can also be observed that an increased number of filters (or increased width of the CNN) helps in improving the performance of CNN architecture. Case 5 of Table 3 also demonstrates the capability of multiple filters in extracting the full features of the handwritten images.

The recognition accuracy of MNIST handwritten digits with different optimizers is shown in Table 4. The optimizers like stochastic gradient descent with momentum (SGDm), Adam, Adagrad and Adadelta are used in the present work to obtain optimized performance. The highest accuracy is achieved using CNN_3L architecture with an Adam optimizer. The Adam optimizer computes adaptive learning rates for each parameter and performs fast convergence. It can be observed that training with the optimizer increases the accuracy of the classifier in both cases involving CNN_3L and CNN_4L. Furthermore, the optimized CNN variant having four layers has less accuracy than the similar variants with three layers. The increased number of layers might cause overfitting and consequently can influence the recognition accuracy. The problem of the overfitting can be avoided by finding out optimal values using trial and error or under some guidance. The concept of dropout may be used to solve the problem of overfitting, in which we can stop some randomly selected neurons (both hidden and visible) from participating in the training process. Basically, dropout is a weight regularization technique and is most preferred in larger networks to achieve better outcomes. Generally, a small value of dropout is preferred; otherwise, the network may be under learning.

The objective of the present work is to thoroughly investigate all the parameters of CNN architecture that deliver best recognition accuracy for a MNIST dataset. Overall, it has been observed that the proposed model of CNN architecture with three layers delivered better recognition accuracy of 99.89% with the Adam optimizer.

The comparison of the proposed CNN-based approach with other approaches for handwritten numeral recognition is provided in Table 5. It can be observed that our CNN model outperforms the various similar CNN models proposed by various researchers using the same MNIST benchmark dataset. Some researchers used ensemble CNN architectures for the same dataset to improve their recognition accuracy but at the cost of increased computational cost and high testing complexity. The proposed CNN model achieved recognition accuracy of 99.89% for the MNIST dataset even without employing ensemble architecture.

## 6. Conclusions

In this work, with the aim of improving the performance of handwritten digit recognition, we evaluated variants of a convolutional neural network to avoid complex pre-processing, costly feature extraction and a complex ensemble (classifier combination) approach of a traditional recognition system. Through extensive evaluation using a MNIST dataset, the present work suggests the role of various hyper-parameters. We also verified that fine tuning of hyper-parameters is essential in improving the performance of CNN architecture. We achieved a recognition rate of 99.89% with the Adam optimizer for the MNIST database, which is better than all previously reported results. The effect of increasing the number of convolutional layers in CNN architecture on the performance of handwritten digit recognition is clearly presented through the experiments.

The novelty of the present work is that it thoroughly investigates all the parameters of CNN architecture that deliver best recognition accuracy for a MNIST dataset. Peer researchers could not match this accuracy using a pure CNN model. Some researchers used ensemble CNN network architectures for the same dataset to improve their recognition accuracy at the cost of increased computational cost and high testing complexity but with comparable accuracy as achieved in the present work.

In future, different architectures of CNN, namely, hybrid CNN, viz., CNN-RNN and CNN-HMM models, and domain-specific recognition systems, can be investigated. Evolutionary algorithms can be explored for optimizing CNN learning parameters, namely, the number of layers, learning rate and kernel sizes of convolutional filters.

## Figures and Tables

**Table 1:** Shallow neural network vs deep neural network.

Factors	Shallow Neural Network (SNN)	Deep Neural Network (DNN)
**Number of hidden layers**	- single hidden layer (need to be fully connected).	- multiple hidden layers (not necessarily fully connected).
**Feature Engineering**	- requires a separate feature extraction process. - some of the famous features used in the literature include local binary patterns (LBPs), histogram of oriented gradients (HOGs), speeded up robust features (SURFs), and scale-invariant feature transform (SIFT).	- supersedes the handcrafted features and works directly on the whole image. - useful in computing complex pattern recognition problems. - can capture complexities inherent in the data.
**Requirements**	- emphasizes the quality of features and their extraction process. - networks are more dependent on the expert skills of researchers.	- able to automatically detect the important features of an object (here an object can be an image, a handwritten character, a face, etc.) without any human supervision or intervention.
**Dependency on data volume**	- requires small amount of data.	- requires large amount of data.

**Table 2:** Configuration details and accuracy achieved for convolutional neural network with three layers.

Model	Layer	k	s	d	p	i/p	o/p	r	Recognition Accuracy (%) and Total Time Elapsed					
									8-16-32	6-12-24	12-24-32	8-16-24	8-24-32	12-24-28
**Case 1**	Layer 1	5	2	2	2	28	14	5	93.76% (20 s)	84.76% (42 s)	98.76% (45 s)	94.08% (46 s)	96.12% (42 s)	98.08% (44 s)
	Layer 2	5	2	1	2	14	7	9						
	Layer 3	5	2	1	2	7	4	25						
**Case 2**	Layer 1	5	2	1	2	28	14	5	96.04% (37 s)	88.91% (27 s)	99% (37 s)	93.80% (37 s)	96.12% (37 s)	98.48% (17 s)
	Layer 2	3	2	1	2	14	7	9						
	Layer 3	3	2	1	2	7	4	17						
**Case 3**	Layer 1	5	2	1	2	28	14	5	98.96% (27 s)	86.88% (27 s)	**99.7%** **(29 s)**	98.72% (39 s)	99.28% (31 s)	99.60% (53 s)
	Layer 2	5	2	1	2	14	7	13						
	Layer 3	5	2	1	2	7	4	29						
**Case 4**	Layer 1	3	3	1	1	28	10	3	80.16% (48 s)	68.40% (29 s)	87.72% (29 s)	78.84% (29 s)	85.96% (51 s)	88.16% (29 s)
	Layer 2	3	3	1	1	10	4	9						
	Layer 3	3	3	1	1	4	2	27						
**Case 5**	Layer 1	5	3	1	2	28	10	5	87.08% (52 s)	80.96% (30 s)	90.08% (24 s)	87.22% (24 s)	92.24% (24 s)	93.32% (24 s)
	Layer 2	3	3	1	1	10	4	11						
	Layer 3	3	3	1	1	4	2	29						
**Case 6**	Layer 1	5	3	1	2	28	10	5	96.48% (23 s)	87.96% (23 s)	97.16% (23 s)	93.68% (24 s)	97.04% (24 s)	98.06% (24 s)
	Layer 2	5	3	1	2	10	4	17						
	Layer 3	5	3	1	2	4	2	53						

**Table 3:** Configuration details and accuracy achieved for convolutional neural network with four layers.

Model	Layer	k	s	d	p	i/p	o/p	r	Recognition Accuracy (%) and Total Time Elapsed				
									6-12-24-32	12-24-24-32	12-24-24-24	6-24-24-24	12-24-28-32
**Case 1**	Layer 1	3	2	1	1	28	14	3	95.36% (56 s)	98.34% (31 s)	98.48% (53 s)	96.56% (44 s)	98.80% (31 s)
	Layer 2	3	2	1	1	14	7	7					
	Layer 3	3	2	1	1	7	4	15					
	Layer 4	3	2	1	1	4	2	31					
**Case 2**	Layer 1	3	2	2	2	28	14	5	84.20% (26 s)	93.72% (30 s)	91.56% (24 s)	89.96% (26 s)	95.16% (25 s)
	Layer 2	3	2	2	2	14	7	13					
	Layer 3	3	2	1	1	7	4	21					
	Layer 4	3	2	1	1	4	2	37					
**Case 3**	Layer 1	5	2	2	2	28	14	9	77.16% (32 s)	94.60% (25 s)	90.88% (26 s)	85.48% (25 s)	94.04% (26 s)
	Layer 2	3	2	2	2	14	7	17					
	Layer 3	3	2	1	1	7	4	25					
	Layer 4	3	2	1	1	4	2	41					
**Case 4**	Layer 1	5	1	2	2	28	17	9	98.20% (30 s)	99.12% (29 s)	99.44% (25 s)	99.04% (22 s)	99.60% (29 s)
	Layer 2	3	2	2	2	17	7	13					
	Layer 3	3	2	1	1	7	4	17					
	Layer 4	3	2	1	1	4	2	25					
**Case 5**	Layer 1	5	1	2	2	28	28	9	98.60% (27 s)	99.64% (27 s)	99.64% (27 s)	99.20% (27 s)	**99.76%** **(43 s)**
	Layer 2	5	2	1	2	28	14	13					
	Layer 3	3	2	1	1	14	7	17					
	Layer 4	3	2	1	1	7	4	27					

**Table 4:** Recognition accuracy with different optimizers.

Model	Recognition Accuracy (%)			
	Momentum (Sgdm)	Adam	Adagrad	Adadelta
CNN_3L	99.76%	99.89	98.67	99.77
CNN_4L	99.76%	99.35	98	99.73

**Table 5:** Comparison of proposed CNN architecture for numeral recognition with other techniques.

Handwritten Numeral Recognition				
Reference	Approach	Database	Features	Accuracy (%)/Error Rate
[75]	CNN	MNIST	Pixel based	0.23%
[76]	CNN	MNIST	Pixel based	0.19%
[8]	CNN	MNIST	Pixel based	0.53%
[77]	CNN	MNIST	Pixel based	0.21%
[78]	CNN	MNIST	Pixel based	0.17%
[79]	Deep Learning	The Chars74K	Pixel based	88.89% (GoogleNet) 77.77% (Alexnet)
[43]	CNN	Urdu Nasta’liq handwritten dataset (UNHD)	Pixel and geometrical based	98.3%
Proposed approach	CNN	MNIST	Pixel and geometrical based	99.89%
